# Supplementary material for: Global Survey of the Full-Length Cabbage Transcriptome (Brassica oleracea Var. capitata L.) Reveals Key Alternative Splicing Events Involved in Growth and Disease Response
Source: Int J Mol Sci. 2021 Sep 28;22(19):10443. doi: 10.3390/ijms221910443 (PMC8508790; doi:10.3390/ijms221910443)
Supplement: Supplementary file 1 [file ijms-22-10443-s001.zip › ijms-1378078-supplementary.pdf]

Supplementary information

Table S1. Summary statistics of circular consensus sequencing (CCS) reads.

Sample	CCS	5'-primer	3'-primer	Poly-A	Full length	FLNC	Average FLNC read length	Consensus reads
flower	318400	299898	302846	295493	233481	223333	2108	113545
fruit	426733	403791	408087	395756	240045	225834	989	119533
root	465686	434505	440723	436358	390619	381672	1682	177861
stem	445709	399511	410518	407923	360440	352724	2720	164425
leaf	260986	243089	244067	220238	200027	197655	2356	93987

Table S2. Statistics of corrected consensus reads.

Sample	Type	Total nucleotides	Total number	Mean length	Min length	Max length	N50	N90
flower	Before correct	225,296,240	113,545	1,985	155	16,407	3,637	966
flower	After correct	227,681,700	113,545	2,006	155	16,183	3,688	976
fruit	Before correct	136,911,757	119,533	1,146	140	16,543	2,635	361
fruit	After correct	129,807,796	83,923	1,547	213	16,551	2,906	550
root	Before correct	304,644,225	177,861	1,713	166	16,797	2,402	874
root	After correct	304,947,061	169,264	1,802	215	14,997	2,439	908
stem	Before correct	452,639,577	164,425	2,753	152	17,192	3,226	1771
stem	After correct	454,707,450	161,417	2,817	262	17,197	3,266	1790
leaf	Before correct	218,581,084	93,987	2,326	173	17,307	3,659	1132
leaf	After correct	219,601,306	90,515	2,427	243	17,338	3,733	1170
